# Modified Gravity: From Black Holes Entropy to Current Cosmology, 4th Edition

**DOI:** 10.3390/e28020222

**Published:** 2026-02-14

**Authors:** Kazuharu Bamba

**Affiliations:** Faculty of Symbiotic Systems Science, Fukushima University, Fukushima 960-1296, Japan; bamba@sss.fukushima-u.ac.jp

Recent cosmological observational data—such as type Ia supernovae (SNe Ia) [1,2,3], cosmic microwave background (CMB) radiation [4,5,6,7,8,9,10,11], the cosmic large-scale structure (LSS) [12,13,14], baryon acoustic oscillations (BAOs) [15,16,17,18], and the effect of weak lensing [19,20,21,22,23]—have led researchers to confirm that, in addition to inflation in the early universe [24,25,26,27] (for recent reviews, see Refs. [28,29,30] and references therein), the late-time expansion of the universe is accelerating.

There are two representative ways of accounting for the late-time cosmic acceleration in the spatially flat universe. The first is to explore the so-called (unknown) dark energy with negative pressure in general relativity. The second is to introduce the extensions of general relativity at large scales, that is, to regard dark energy as a geometrical component. In regard to the second approach, a number of alternative theories of gravity have been proposed (for comprehensive reviews, see, for example, Refs. [31,32,33,34,35,36,37,38,39,40,41,42,43,44,45,46,47,48,49,50,51,52,53,54,55,56,57,58,59,60,61,62,63,64,65,66,67,68,69,70,71]).

This Special Issue of *Entropy*, “Modified Gravity: From Black Holes Entropy to Current Cosmology, 4th Edition”, contains eight original research studies on modern astrophysics and cosmology. It is arranged as follows. In the first part, black holes [72,73,74,75] are investigated. In the second part, topics relating to cosmology [76,77,78,79] are explored. A brief overview of each article is given below.

A hot NUT–Kerr–Newman black hole is studied in Ref. [72]. The corrections for both the Hawking temperature and Bekenstein–Hawking entropy are discussed in detail through the semi-classical approach.

In Ref. [73], a hyperbolically symmetric black hole is investigated to explore how it differs from conventional classical black holes via an analysis of the dynamics of test particles around the black hole.

In Ref. [74], the modifications of entropy for charged accelerating Anti-de Sitter black holes are discussed based on correction with the gauge invariance originating from an analysis of the quantum field equations with the violations of the Lorentz symmetry for curved spacetime.

A rotating Kiselev black hole is considered in Ref. [75]. Using several approaches, such as the WKB approximation and the effect of quantum-tunneling radiation, the authors explore whether a black hole is surrounded by a quintessence field, playing the role of a dark energy component responsible for late-time cosmic acceleration.

The dynamics of the late-time universe are discussed in Ref. [76] to obtain clues useful for resolving the Hubble tension. As the background gravity theory, f(R) gravity is introduced in the metric formalism, and the process of the creation of matter is examined.

In Ref. [77], two kinds of late-time cosmology are studied to explore the deviation from the standard ΛCDM model: (1) a dynamical dark energy originating from the gravitational field in the expanding universe and (2) a model of the interaction between the dark energy component and matter.

In Ref. [78], a cyclic universe scenario with cosmic contraction and expansion is proposed in the context of the quantum memory matrix cosmology, in which it is assumed that the spacetime is constructed by finite-capacity Hilbert cells.

The authors of Ref. [79] discuss thermodynamics in quasi-de Sitter spacetime, focusing on a specific case where the energy-momentum tensor of spacetime first reproduces a quintessence field asymptotically. A finite domain, in which an additional Cauchy horizon appears, is then analyzed.

We find it very likely that the eight articles summarized above will be useful for future related studies on various aspects of modern cosmology and astrophysics. 
